# Comparative Analysis of Gut Microbiota of Native Tibetan and Han Populations Living at Different Altitudes

**DOI:** 10.1371/journal.pone.0155863

**Published:** 2016-05-27

**Authors:** Kang Li, Zeng Dan, Luobu Gesang, Hong Wang, Yongjian Zhou, Yanlei Du, Yi Ren, Yixiang Shi, Yuqiang Nie

**Affiliations:** 1 Department of Gastroenterology, Guangzhou First People’s Hospital of Guangzhou Medical University, Guangzhou, 510180, Guangdong Province, China; 2 High Altitude Medical Research Institute, People’s Hospital of Tibet Autonomous Region, Lhasa, 850000, China; 3 Shanghai Major Bio-pharm Technology Co., Ltd., Shanghai, 201203, China; Indiana University, UNITED STATES

## Abstract

The factors driving the composition of gut microbiota are still only partly understood but appear to include environmental, cultural, and genetic factors. In order to obtain more insight into the relative importance of these factors, we analyzed the microbiome composition in subjects of Tibetan or Han descent living at different altitudes. DNA was isolated from stool samples. Using polymerase chain reaction methodology, the 16S rRNA V1–V3 regions were amplified and the sequence information was analyzed by principal coordinates analysis and Lefse analyses. Contrasting the Tibetan and Han populations both living at the 3600 m altitude, we found that the Tibetan microbiome is characterized by a relative abundance of *Prevotella* whereas the Han stool was enriched *in Bacteroides*. Comparing the microbiome of Han stool obtained from populations living at different altitudes revealed a more energy efficient flora in samples from those living at higher altitude relative to their lower-altitude counterparts. Comparison of the stool microbiome of Tibetan herders living at 4800 m to rural Tibetans living at 3600 m altitude shows that the former have a flora enriched in butyrate-producing bacteria, possibly in response to the harsher environment that these herders face. Thus, the study shows that both altitude and genetic/cultural background have a significant influence on microbiome composition, and it represents the first attempt to compare stool microbiota of Tibetan and Han populations in relation to altitude.

## Introduction

As the largest and most complex micro-ecosystem of the body, the gut microbiota and their metabolites play extremely important roles in protecting the intestinal mucosal barrier and thus maintaining human health [1–6]. In accordance with their function in aiding food digestion, the gut microbiota genome appears significantly enriched in genes that participate in the metabolism of carbohydrates, amino acids, vitamins, and short-chain fatty acids, and therefore, acts to maintain normal physiological and metabolic functions of the body [3, 4]. The micro-organisms of the gut microbiota are in a long-term mutually beneficial symbiosis. This balanced and stable intestinal microenvironment system plays a crucial role in the adjustment of hosts to the special conditions. Although human gut microbiota are largely similar among various populations, differences in the species and/or strain compositions do exist. Hosts’ geographical environment, gender, age, diet, lifestyle, physical and psychological state, and health status have all been reported to influence the composition of gut microbiota[7–10]. Particularly, dietary pattern is one of the important factors affecting the gut microbiota composition [11–14].

Native Tibetans living on the Qinghai-Tibet plateau (more than 3000 m above sea level), have gradually adapted to the special plateau environment which is characterized by low oxygen and low air pressure, due to its high altitude[15], and these people have developed unique genetic predispositions, lifestyles, and dietary habits[16]. In most pastoral areas, especially at high altitude, meat (beef and mutton), Yak butter, milk, and other dairy products account for the major proportion of the diet in conjunction with a high sodium intake from Tibetans’ food sources, while consumption of vegetables and fruits is significantly less than that in the low-altitude population. For Tibetans, especially Tibetan farmers, a daily staple is roasted barley flour, which has highland barley as its main ingredient, while their common fluid consumption are teas, such as Yak butter and salt tea [16, 17]. How this specific diet influences the gut microbiome has not been established yet.

It has been demonstrated that a close relationship exists between the gut microbiota and various health problems [18–20]. The exact factors driving microbiome composition remain only partially understood, but involve environmental, genetic and life-style factors. The relative importance of these factors remains largely obscure. However, comparison of the microbiome in groups from similar descent but living in different environment, or groups from a different genetic and cultural background but living in the same environment may provide important answers here. To date, although the gut microbiota across seven Chinese ethnic groups, including Tibetan and Han low-altitude populations, have been characterized [21, 22], Tibetan and Han populations, however, living at different altitudes have not been studied, and little is known about the correlations between the composition of gut microbiota and environmental factors, genetic backgrounds, lifestyle characteristics, and dietary habits of Tibetans at different altitudes. Hence, in this study, we performed a comparative analysis of the relative abundances of various micro-organisms in the gut microbiota of Tibetan and Han populations living at different altitudes.

## Materials and Methods

### Ethics statement

The experimental protocol was established according to the ethical guidelines of the Helsinki Declaration and approved by the ethics committee of People's Hospital of Tibet Autonomous Region, Lasha, China. Written informed consent was obtained from the individual participants.

### Subjects

The subjects belong to four groups: 1) 13 native Tibetan herders living at an altitude of more than 4800 m; 2) 13 native Tibetan peasants living at an altitude of 3600 m; 3) 12 individuals of the Han population who migrated to a high altitude and lived in Lhasa (3600 m) for over 20 years; and 4) 30 citizens of a low-altitude Han population who lived in the Chinese hinterland (Chengdu, Sichuan Province) at an altitude of about 500 m. All of the enrolled subjects were 35–55 years old, had normal weight (body mass index = 19–24 kg/m^2^) and were healthy without a history of gastrointestinal disease, liver disease, hypertension, or diabetes, as demonstrated by their medical histories and physical examinations (S1 Table). None of the enrolled subjects took any antibiotic or microbial modulator within 2 months before sampling. All of the native Tibetans followed the traditional lifestyle and dietary habits of their ethnic groups. That means, for the Tibetan herders, that their daily food intake is mostly meat- and milk-based, and for the Tibetan peasants, the diets have higher percentages of vegetable and fruits than their herders counterparts. For Han populations who migrated into and lived in Lhasa for over 20 years, their dietary habits were still basically the same as those of Han living in other parts of China. All of the enrolled subjects were adequately informed about the sampling process and research protocols before sampling and signed an informed consent form.

### Stool sampling

Fresh stool samples were collected and transferred to the laboratory. Their 200-mg samples were placed in new 2-mL sterile centrifuge tubes, quickly placed on ice, and transferred into a -70°C cryogenic freezer for cryopreservation. The entire sampling process was finished within 30 minutes.

### DNA extraction

**S**amples were first mechanically disrupted by glass beads, and then DNA was extracted using the E.Z.N.A Stool DNA Kit (Omega, USA) according to the manufacturer’s instructions. DNA (3 μl) was run on a 1% agarose gel to detect the size and integrity of DNA fragments. The quantity and quality of DNA were determined using a NanoDrop ND-1000 (ThermoFisher, USA), and DNAs with an A260/280 ratio of 1.8–2.0 were used for subsequent polymerase chain reaction (PCR) amplification.

### PCR amplification of 16S rRNA V1–V3 regions

The V1–V3 region (27F-533R) of 16S rRNA was amplified by PCR with the primers 27F (5'-AGAGTTTGATCCTGGCTCAG-3') and 533R (5'-TTACCGCGGCTGCTGGCAC-3'). The PCR components were as follows: 5× FastPfu Buffer (4 μl), 2.5 mM dNTPs (2 μl), forward primer (5 μM, 0.8 μl), reverse primer (5 μM, 0.8 μl), FastPfu polymerase (0.4 μl), and template DNA (10 ng). The reaction volume was brought up to 20 μl with ddH_2_O. The PCR conditions were 2 minutes at 95°C followed by 30 cycles of 30 seconds at 95°C, 30 seconds at 55°C, and 45 seconds at 72°C, and finally 10 minutes at 72°C.

### High-throughput sequencing

Roche 454 (Roche, Switzerland) high-throughput sequencing of the PCR products was performed by Shanghai Majorbio Biological Technology Co. Ltd., Shanghai, China.

### Bioinformatics analysis

For quality control of the raw data the following criteria were applied:

1. The number of mismatches within a primer that was found in the first sequencing had to be less than two.
2. The primer and adapter sequence of the 3' end of each read was trimmed within the parameter “maximum number of mismatches = 3”.
3. The base quality test window that was shifted at one-base pair step width was set at 50 bp in length. When the average quality in the window was lower than 20, the preceding sequence was intercepted from this position.
4. Reads with ambiguous bases or a length of high repeat (homologous) regions of a single base of more than 10 bp or less than 200 bp in length were discarded.
5. Chimeric sequences generated during the PCR amplification were detected and disposed of by the uchime method [23].

OTU clustering analysis was performed at 97% similarity. A naive Bayesian classifier was applied for the assignment of 16S rRNA sequences into the bacterial taxonomy [24] with a confidence threshold set at 70%, and the SILVA [25] database was employed for training. The calculations of the rarefaction curve, richness index, diversity index, Bray–Curtis distance and principal coordinates analysis were performed with the mother [26] built-in commands. The Lefse software[27], which performs a nonparametric Wilcoxon sum-rank test followed by linear discriminant analysis (LDA) coupled with measurements to assess the effect size of each differentially abundant taxon, was used to search for taxon for which the relative abundance was significantly different among the various populations. An alpha = 0.05 was used in Wilcoxon rank sum test, and the log value for the LDA analysis was set to be less than 2.0.

## Results

### 454 sequencing

**Fig 1:**
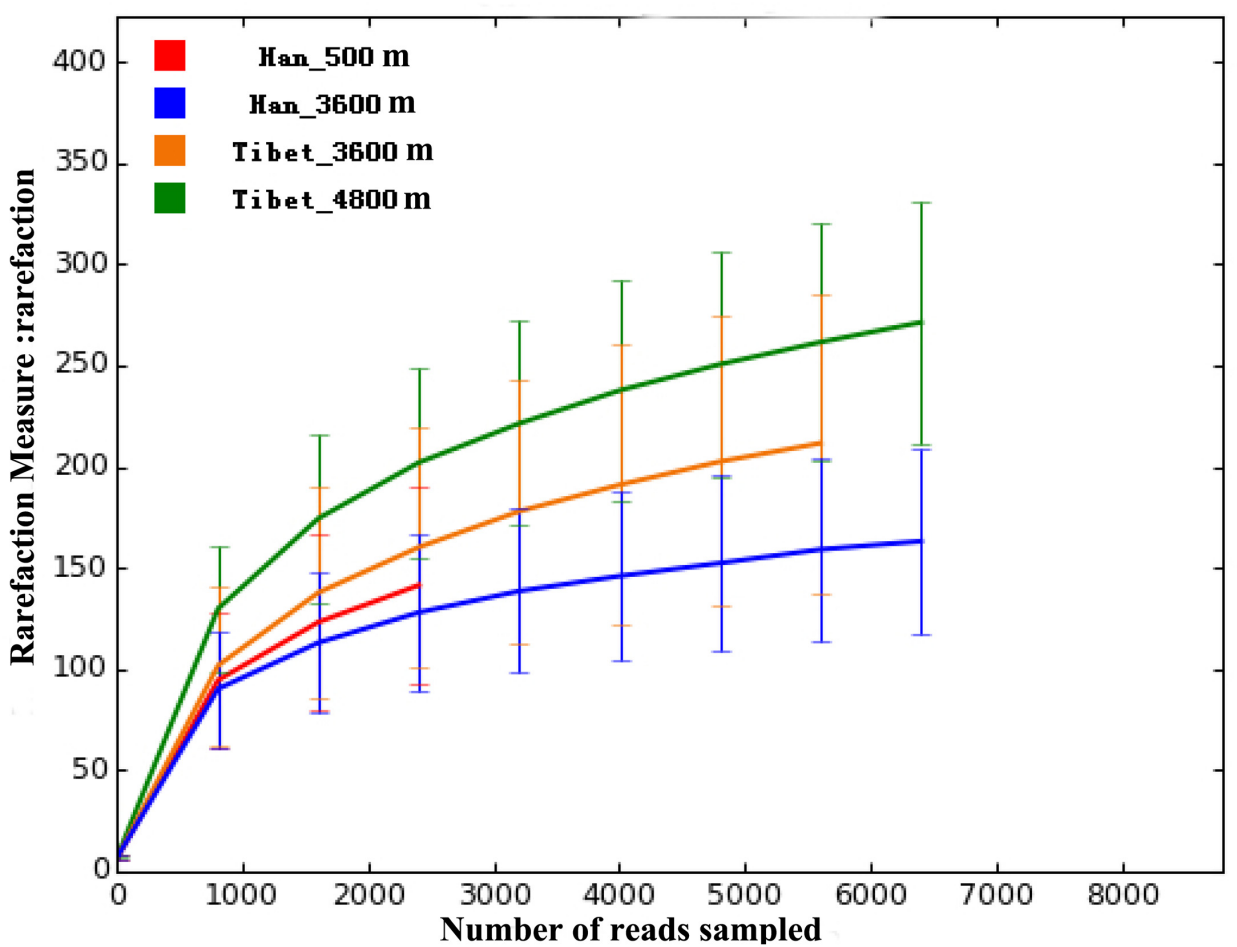
Rarefaction curves, with error bars showing confidence intervals, for all four groups of samples based on OTUs detected using a similarity threshold of 97% (0.97).

Based on the 454 sequencing platform, all of the polymerase chain reaction (PCR) products from 16S rRNA V1–V3 regions of stool DNA from 38 subjects (13 native Tibetan herders living at an altitude of more than 4800 m, 13 native Tibetan peasants living at an altitude of 3600 m, and 12 Han individuals living on the plateau at 3600 m) were sequenced. The total amount of data was 359,385 reads, with an average of 9457 reads per sample and a median read length of 446 bp. For the 30 samples of low-altitude Han individuals, the total amount of data included 301,756 reads, with an average of 10,058 reads per sample and a median read length of 446 bp. By clustering analysis at a 97% similarity, 859 operational taxonomic units (OTUs) were identified in total. Rarefaction curves are shown in Fig 1. No significant difference in either OTU abundance (ace, chao1 index) or OTU diversity index (Simpson, Shannon index) was observed between the Tibetan and Han populations.

### Taxonomic analysis

**Fig 2:**
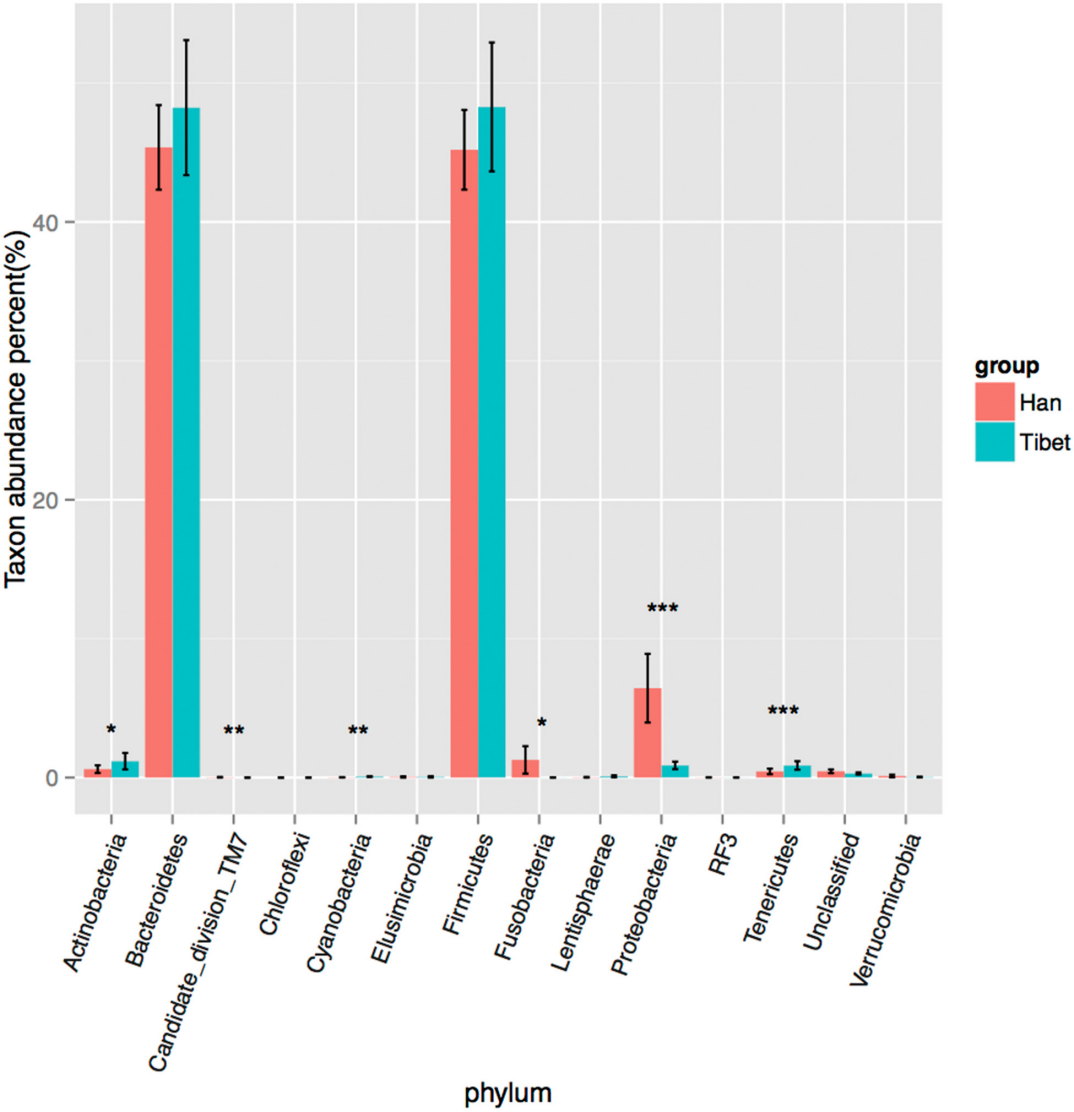
Relative bacterial abundance in the gut microbiota of the Han and Tibetan populations at the phylum level. *represents p<0.05, **represents p<0.01, ***represents p<0.001.

**Fig 3:**
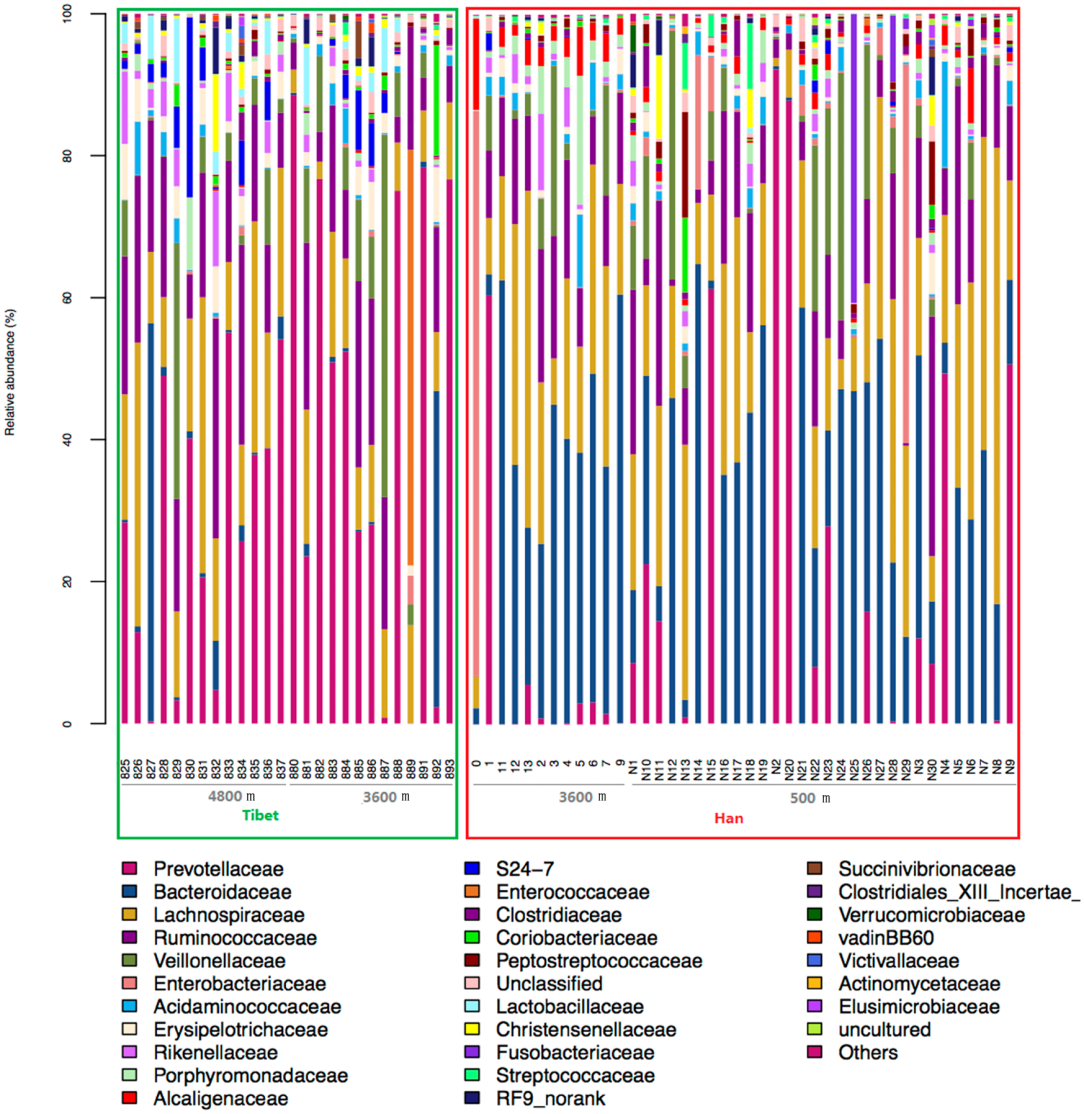
Relative bacterial abundance at the family level for all gut microbiota samples. The Tibetan samples are included in green box, and the Han samples in red box.

The OTU species classification was carried out by comparative analysis with the SILVA database. In both Tibetan and Han groups, the relative abundances of *Bacteroidetes and Firmicutes* were the highest, accounting for more than 90% of all species (Fig 2). The relative abundance of *Tenericutes* in Tibetans was higher than that in the Han population (p<0.001, Bonferroni-corrected Mann–Whitney U test), whereas the *Proteobacteria* abundance appeared to be higher in the Han populations than that in Tibetans (p<0.001, Bonferroni-corrected Mann–Whitney U test). The relative abundance of *Actinobacteria* in Tibetans was slightly higher than that in the Han population (p<0.05, Bonferroni-corrected Mann–Whitney U test), while the *Fusobacteria* abundance appeared to be higher in the Han populations (p<0.05, Bonferroni-corrected Mann–Whitney U test). A larger difference between both groups in the species distribution was found at the family level. The number of species of *Prevotellaceae* in Tibetans was higher than that in the Han population (36.58% vs 12.73%, p<0.05, Bonferroni-corrected Mann–Whitney U test), whereas that of *Bacteroidaceae* in the Han population was higher than that in the Tibetan (29.24% vs 4.74%, p<0.05, Bonferroni-corrected Mann–Whitney U test; Fig 3). These results suggest a remarkable difference between Tibetans and the Han population in terms of the species composition of their gut microbiota, demonstrating the importance of genetic and cultural factors over the environment *per se*.

### Beta diversity analysis

**Fig 4:**
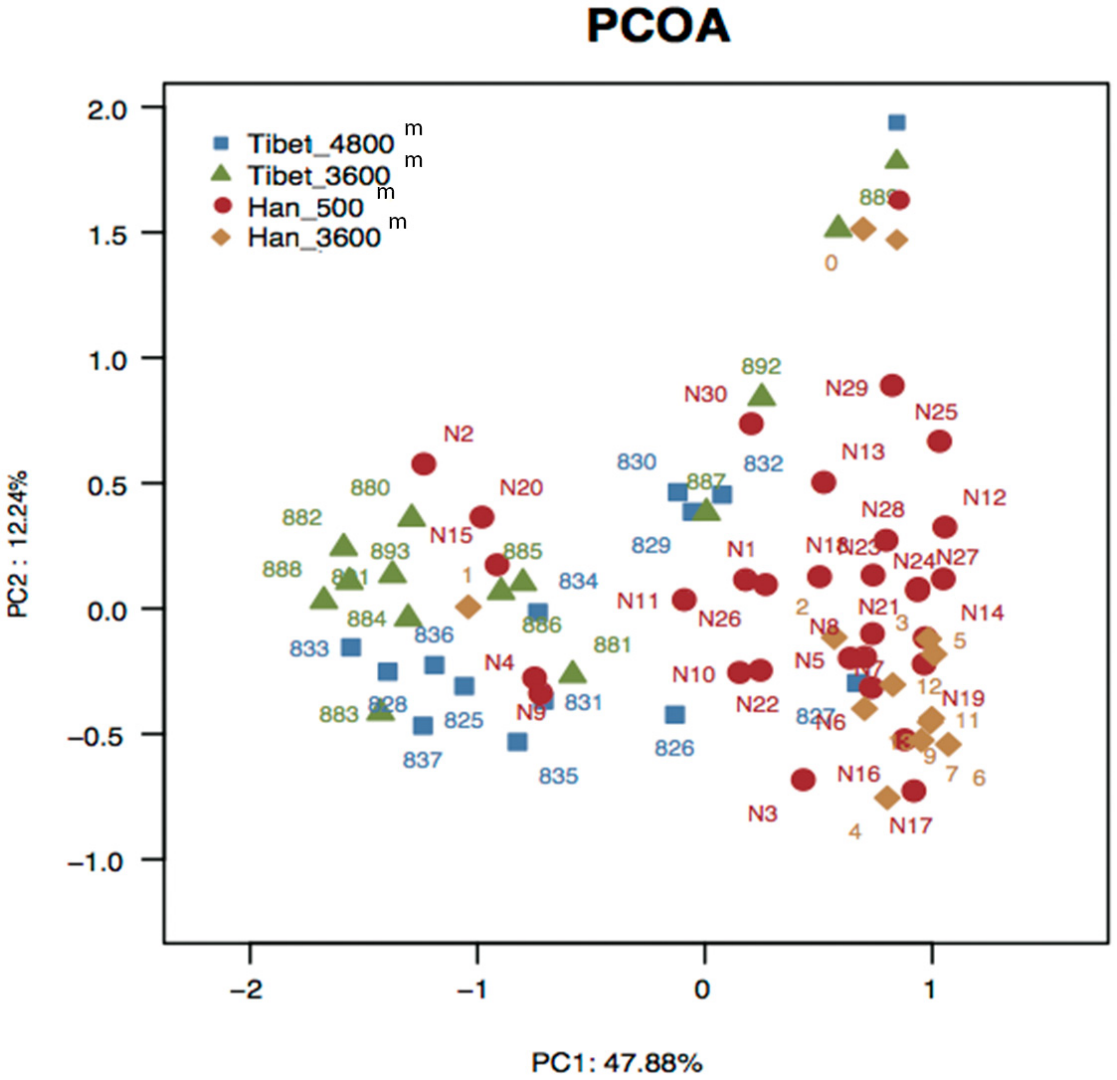
Principal coordinates analysis (PCOA) based on the distance matrix of Bray-Curtis dissimilarity of the microbial community between samples.

To further analyze whether the structure of the bacterial community in the gut microbiota differs between native Tibetans and the Han population, principal coordinates analysis (PCOA) based on the Bray–Curtis distance was performed. The results revealed there were significant differences in gut microbial community structure between the Tibetans and the Han population with regard to the first two principal component scores, which accounted for 47.88% and 12.24% of the total variations. Also, a slight difference exists between Tibetan herders and farmers. However, no obvious separation between the immigrant Han and the low-altitude Han population was observed (Fig 4), thus again highlighting the dominance of genetic and cultural factors, over altitude environment.

There are many factors which may have impacts on the composition of gut microbiota, such as gender, age, diet structure, ethnicity, and other environmental considerations. However, the CH index[28] (as plotted in S1 Fig) indicates that two is the optimal number of clusters, and the RDA plot (S2 Fig) indicates that the two most important contributing factors are altitude and diet. Hence we chose to concentrate on examining the impacts of altitude and ethnicity, as the diets between Han and Tibetan people are considerably different and it is impossible to separate genetic factors from dietary factors in this context.

### Differences in gut microbiota between native Tibetan and Han populations at the same altitude (3600 m)

**Fig 5:**
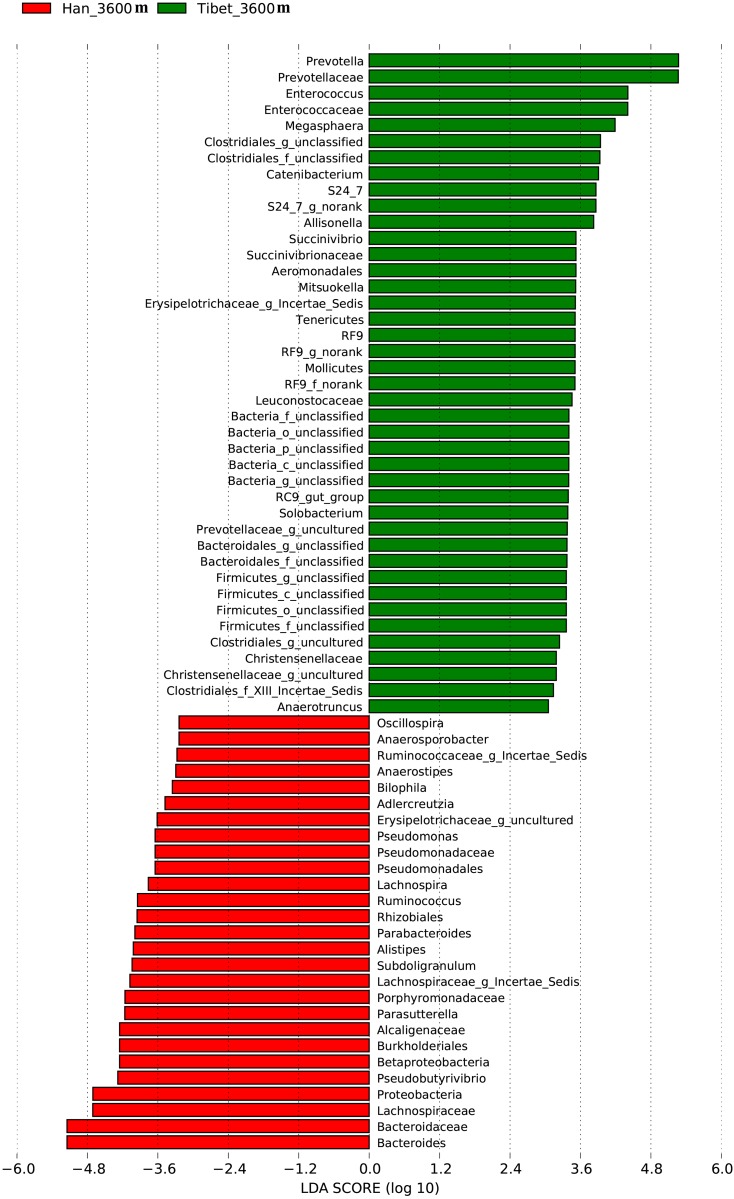
Analysis of differences in the microbiota between the immigrant Han population and native Tibetans living at the same altitude (3600 m) using Lefse software (linear discriminant analysis [LDA] coupled with effect size measurements). Taxa enriched in the Tibetan population are indicated with a positive LDA score (green), and taxa enriched in the immigrant Han population have a negative score (red). Only taxa meeting an LDA significant threshold of 2 are shown. For taxa, which were defined as unclassified, no rank, uncultured or Incertae-Sedis, the name of a higher taxon level was added before its taxon abbreviation. p, phylum; c, class; o, order; f. family; g, genus; s, species.

To study the differences in gut microbiota between native Tibetans and immigrant Han populations living at the same altitude (3600 m), a Lefse difference analysis of taxon abundance was performed. The differences in gut microbiota between native Tibetan and high-altitude immigrant Han populations were thus identified. In native Tibetans, the relative abundances of *Prevotella*, *Prevotellaceae*, *Enterococcus*, *Enterococcaceae*, and *Megasphaera* were higher than those in the immigrant Han population. The abundances of *Bacteroides*, *Bacteroidaceae*, *Lachnospiraceae*, *Proteobacteria*, and *Pseudobutyrivibrio* were higher among the gut microbiota of the immigrant Han population compared to that of the native Tibetans (Fig 5).

### Differences in gut microbiota between Low-altitude Han and high-altitude immigrant Han populations

**Fig 6:**
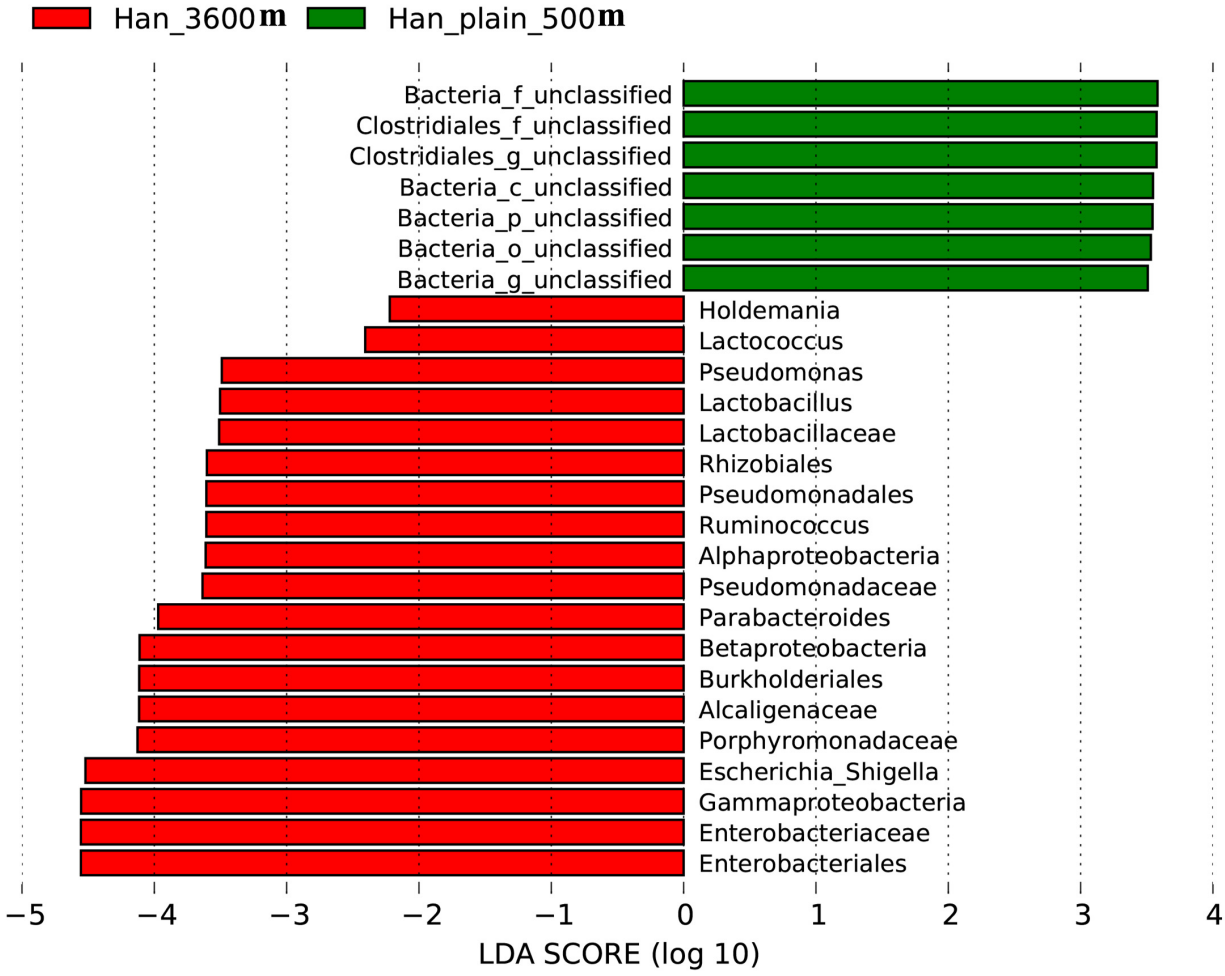
Analysis of differences in the microbiota between the low-altitude Han population and high-altitude immigrant Han population using Lefse software. Taxa enriched in the immigrant Han population are indicated with a positive LDA score (green), and taxa enriched in the low-altitude Han population have a negative score (red). Only taxa meeting an LDA significant threshold of 2 are shown. For taxa, which were defined as unclassified or uncultured, the name of a higher taxon level was added before its taxon abbreviation. p, phylum; c, class; o, order; f. family; g, genus.

The taxonomic abundances in the gut microbiota of the low-altitude Han population living in Chengdu city and the immigrant Han population living in Lhasa city were compared by Lefse analysis. More *Enterobacteriales*, *Enterobacteriaceae*, *Gammaproteobacteria*, *Escherichia Shigella*, and *Porphyromonadaceae* as well as several other types of microbial species are found in the gut microbiota of the immigrant Han population living in Tibet Plateau than in that of the low-altitude Han population (Fig 6).

### Differences in gut microbiota between Tibetan herders and Tibetan peasants

**Fig 7:**
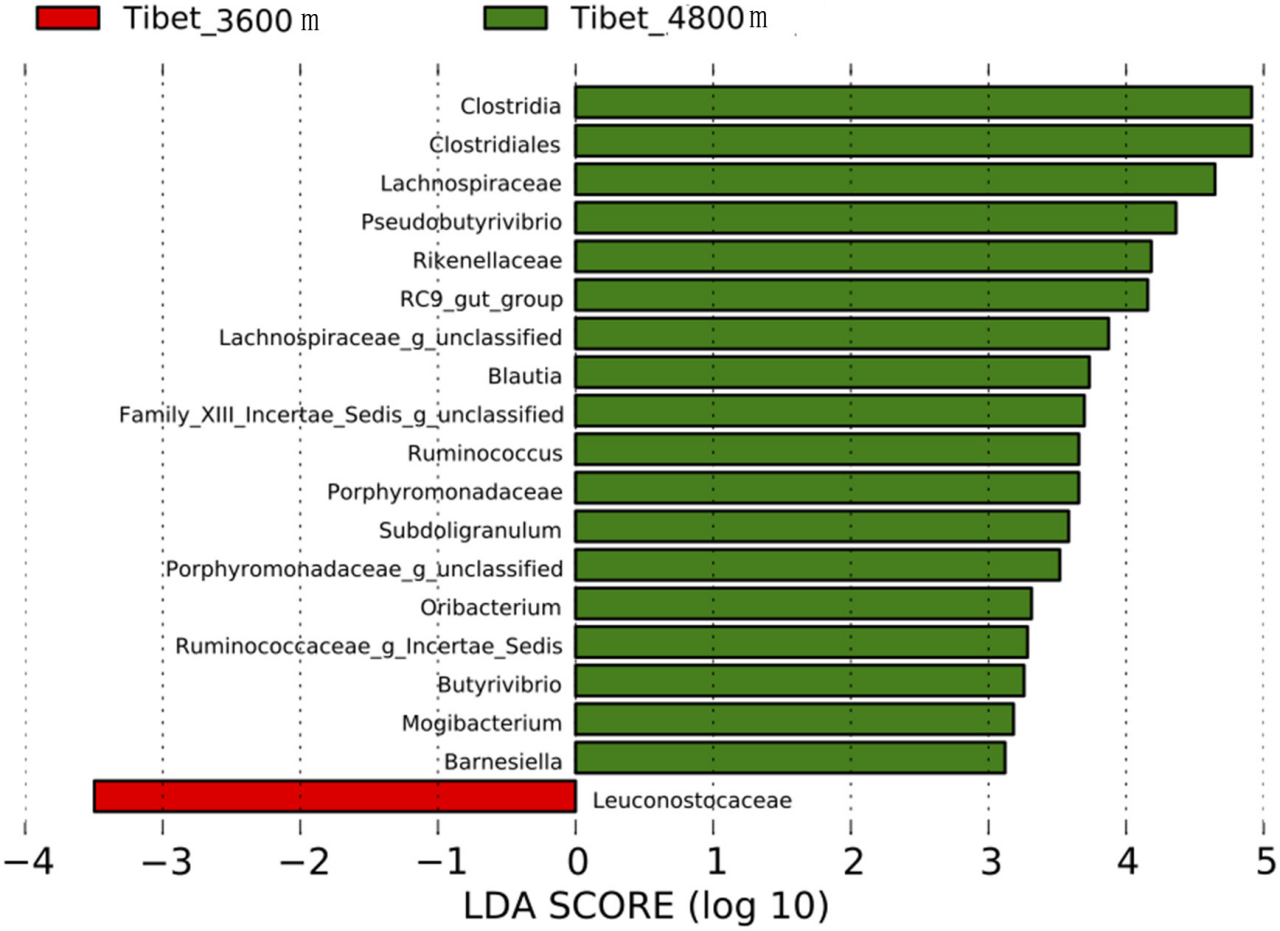
Analysis of differences in the microbiota among Tibetans living at different altitudes using the Lefse software. Taxa enriched in the microbiota of Tibetans living at 4800 m are indicated with a positive LDA score (green), and taxa enriched in Tibetans living at 3600 m have a negative score (red). Only taxa meeting an LDA significant threshold of 2 are shown. For taxa, which were defined as unclassified and Incertae-Sedis, the name of a higher taxon level was added before its taxon abbreviation. g, genus.

The taxonomic abundances in gut microbiota of native Tibetans living at different altitudes were compared by Lefse analysis. Greater proportions of *Clostridia*, *Clostridiales*, *Lachnospiraceae*, *Pseudobutyrivibrio*, and *Rikenellaceae* were found in Tibetan herders living at an altitude of more than 4800 m than in Tibetan peasants living at an altitude of 3600 m. Only the proportion of *Leuconostocaceae* in gut microbiota of Tibetan peasants was greater than that in Tibetan herders (Fig 7).

## Discussion

There is still little insight into the relative importance of the environment, ethnic background and diet with respect to the composition to the microbiome. To obtain more insight into this issue we studied the composition of gut microbiota of Tibetan and Han populations residing at different altitudes. We found significant differences in the species composition of gut microbiota between the Tibetan and immigrant Han populations living at the same altitude, between Han populations living at different altitudes, and between Tibetans living at different altitudes. The relative abundances of *Bacteroidetes* and *Firmicutes* were the highest, accounting for more than 90% of all species at the phylum level. The classification analysis of OTUs in the SILVA database reveals a remarkable difference between the native Tibetans and the Han population in the phylum level of *Tenericutes and Proteobacteria*. Thus our results quantify the strong influence of ethnic background on microbiome composition but also show the influence the environment (in this case altitude) can exert.

**Fig 8:**
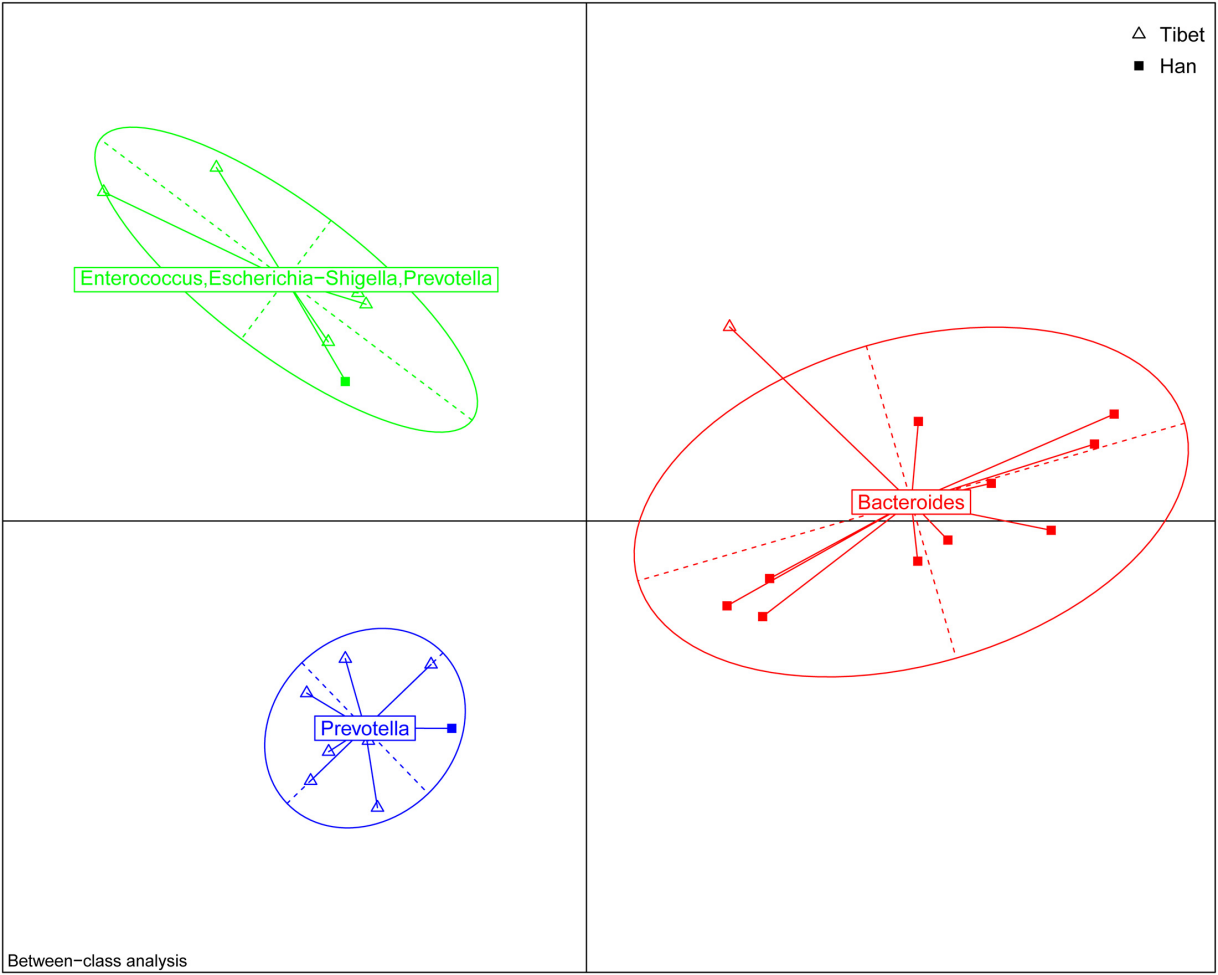
The Enterotype Analysis for Han and Tibetan living at the 3600m altitude. The data indicates that most of the Han belong to the *bacteriodes* type while those of Tibetans leans strongly to the *Prevotella* type.

The analysis for each microbiota group revealed that the abundances of various bacteria, including *Prevotella*, *Prevotellaceae*, *Enterococcus*, *Enterococcaceae*, *Megasphaera*, and *Clostridiales* in native Tibetans were all significantly higher than those in the immigrant Han population living at the same altitude (3600m), while in the latter group, the level of *Bacteroides* was higher. Previous reports have classified the human gut into three enterotypes, and each are respectively dominated by *Prevotella*, *Bacteroides*, and *Ruminococcus* [29, 30]. Our analysis indicates that at the 3600 m level, most Han people belong to the *Bacteroides* enterotype, while most Tibetans are of the *Prevotella* enterotype (Fig 8). The Tibetans’ agricultural lifestyle at high altitude, especially their different dietary habits (high-fiber, low-fat based diet) compared to those of the modern Han city population diet structure (low-fiber, high-animal protein and high-fat based diet) might be the main factor for the Tibetan’s distinct gut microbiota. This finding is consistent with a previous report that the relative abundances of *Prevotella* and *Bacteroides* are negatively correlated [31], with the relative abundance of *Bacteroides* being positively associated with diets rich in animal fat and protein, whereas the *Prevotella* enterotype was associated with low intake values for fat and protein but with high intake of carbohydrates and simple sugars [32].

In present study, when comparing the gut microbiota of the Han population who originally resided in lower altitude regions but then migrated to a higher altitude region versus those of the Han population who live in the lower altitude regions, we found that the former group had greater relative abundances of various bacteria, such as *Enterobacteriales*, *Enterobacteriaceae*, *Lactococcus*, and *Lactobacillus*. Considering that both Han populations basically share the same typical modern Chinese city lifestyle and dietary habits, we speculate that the difference between the two Han populations in the species composition of their microbiota might result from the differences in the living environment at different altitudes, although other dietary and genetic factors are difficult to rule out completely. This may be due to differences in erythropoietin levels in populations living at different altitudes, as it has been shown that erythropoietin suppresses human monocyte function and thus may alter gastrointestinal immunity[33] and hence the composition of bacterial flora. Alternatively, the body may actively adapt to the bacterial flora under altered circumstances, as for instance also happens during pregnancy[34]. Living at a higher altitude is probably harsher and requires stronger assistance from their gut microbiota to support nutrient extraction form the diet. Since most of these bacterial types gained by living at higher altitude help to ferment sugar-based food into nutrients our bodies can absorb, it is conceivable that the microbiome changes observed in the present study constitute an adaptive response in this respect.

Epidemiological investigations of populations with a high or low incidence of colorectal cancer (CRC) and various dietary habits indicated that gut microbiota changes are closely related to the occurrence and development of CRC [35–37]. In our study, the relative abundances of gut microbiota, including *Clostridia*, *Clostridiales*, *Lachnospiraceae*, *Pseudobutyrivibrio*, *Blautia*, and several others, were found to be higher in the microbiota of Tibetans who lived in pastoral areas at an altitude of more than 4800 m than in that of Tibetans who lived in agricultural areas at an altitude of 3600 m. It has been reported that higher abundances of *Clostridiales*, *Clostridium*, and *Lachnospiraceae* relate to a healthier gut, because these bacteria produce short chain fatty acids (SCFA), especially butyrate, which is an important energy source for intestinal epithelial cells and thus plays a key role in maintaining gut homeostasis [38]. Hence, it is tempting to speculate that colonization by these bacteria may be beneficial for the Tibetans living at higher altitude and relate to adaptions to a low-oxygen environment. Furthermore, the production of butyrate is also known to be anti-tumorigenetic [39]. Indeed, *Blautia* has been reported to be underrepresented in the guts of CRC patients [40]. Tibetan herders who live in remote plateau areas at an extremely high altitude of more than 4800 m still lived a traditional nomadic life, and their dietary habits, which are dominated by dried, half-cooked beef and mutton, buttered tea, milk tea, milk, and fermented dairy products such as yogurt, have remained unchanged, whereas neither vegetables nor fruits are common in the diet of these people. As such a diet might be associated with an increased susceptibility to CRC, the presence of these anti-carcinogenic bacteria might be a factor as to why these people suffer from a relatively low incidence to CRC. The relatively pure, native ecology in pastoral areas where the Tibetan lifestyle is less affected by modern industrialization may be beneficial for the presence of species such as *Clostridiales* and *Clostridia* in the gut microbiota of the Tibetans, helping them to maintain a healthier gut environment. On the other hand, some crops, vegetables, and fruits are produced in agricultural areas at an altitude of 3600 m where Tibetans eat significantly less beef, mutton, and dairy products as compared to the Tibetan nomadic population. Therefore, despite a potential effect of oxygen pressure, dietary characteristics may be still a decisive factor explaining the differences in the species composition of gut microbiota between Tibetans living at different altitudes.

Interestingly, levels of two bacteria, *Lachnospiraceae* and *Pesudobutyrivibrio*, were remarkably different in a comparison of the native Tibetans living at 3600 m of altitude to either Tibetans at 4800 m of altitude or the Han population also living at 3600 m of altitude (both were lower in Tibetans at 3600 m altitude). Similar to *Lachnospiraceae*, *Pesudobutyrivibrio* is also capable of producing butyrate [41]. The functional significance of this observation, if any, remains to be elucidated.

In conclusion, to the best of our knowledge, this is the first report of differences in gut microbiota between Tibetan and Han populations living at different altitudes. The results indicated that stratified intestinal microbiota variation in gut bacteria exists among different populations. Our findings may provide some insight for further study of gut microbiota dysbiosis-related diseases in Tibetan and Han populations.

## Supporting Information

S1 Fig Assessment *of Clustering Quality*. The CH index[1] indicates that two is the optimal number of clusters.
(PDF)

S2 Fig RDA analysis. Height and diet appear the two most important contributing factors explaining interindividual variance in gut microbiome.
(PDF)

S1 Table Sampling Information. The gender, age, ethnicity, occupation, location, diet style, BMI and blood oxygen level are listed for the 68 participants.
(DOC)
