# Supplementary figures and images for: Comparative Analysis of Gut Microbiota of Native Tibetan and Han Populations Living at Different Altitudes
Source: PLoS One. 2016 May 27;11(5):e0155863. doi: 10.1371/journal.pone.0155863 (PMC4883765; doi:10.1371/journal.pone.0155863)

# Optimal number of clusters

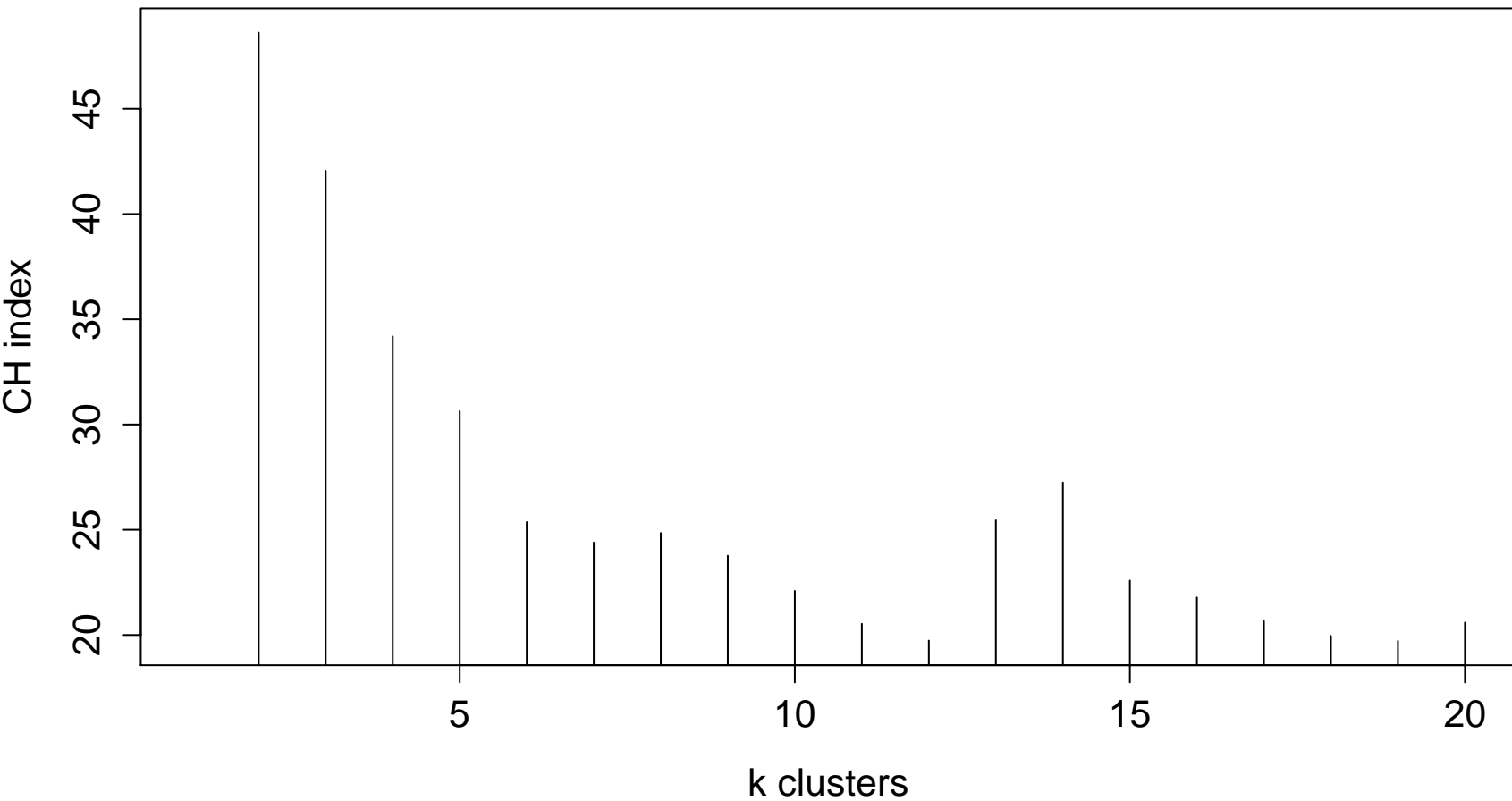

Supplement: S1 Fig — The CH index[1] indicates that two is the optimal number of clusters. (PDF) [file pone.0155863.s001.pdf]

RDA 1 : 6.54%

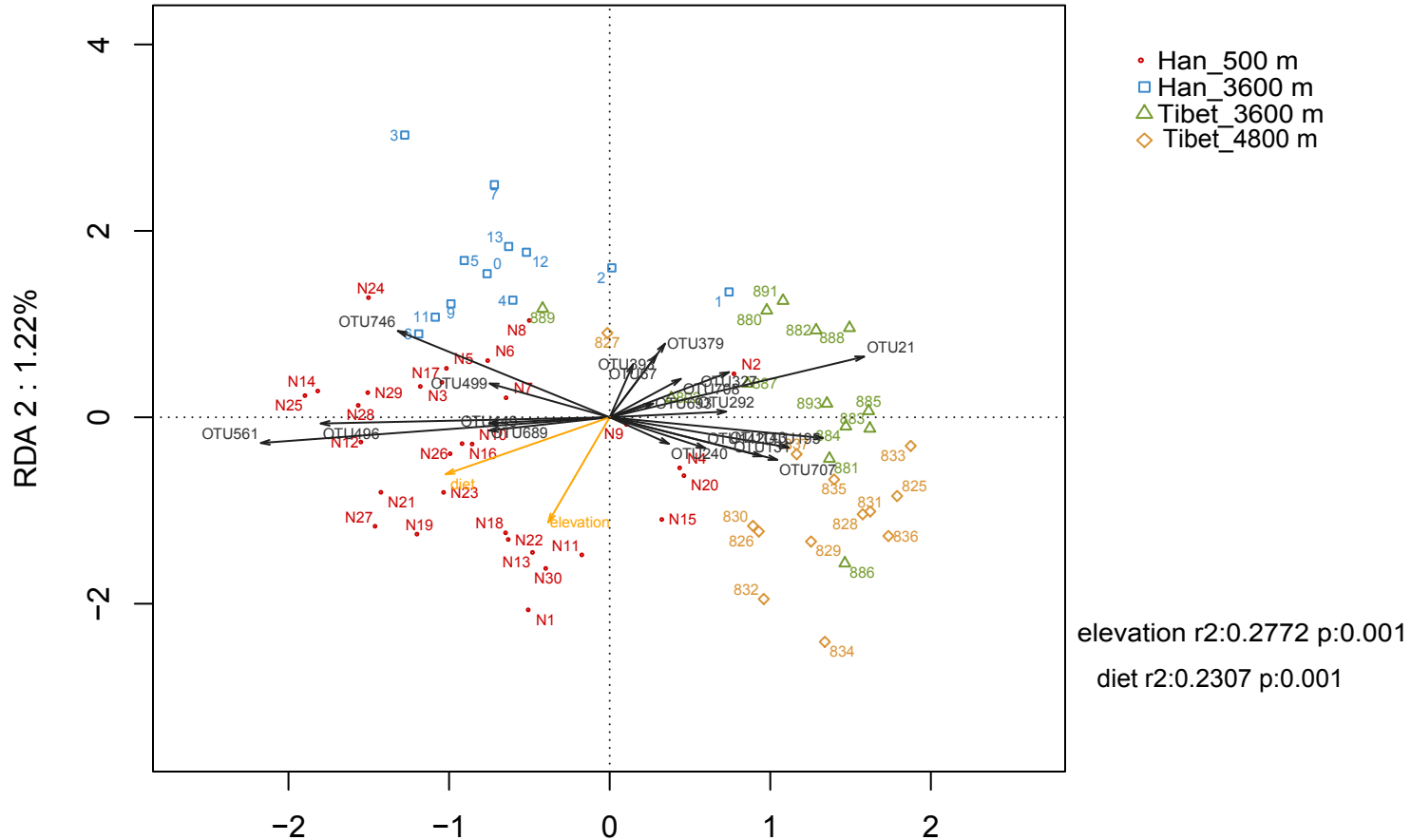

Supplement: S2 Fig — Height and diet appear the two most important contributing factors explaining interindividual variance in gut microbiome. (PDF) [file pone.0155863.s002.pdf]
