# Supplementary material for: Comparative Analysis of Gut Microbiota of Native Tibetan and Han Populations Living at Different Altitudes
Source: PLoS One. 2016 May 27;11(5):e0155863. doi: 10.1371/journal.pone.0155863 (PMC4883765; doi:10.1371/journal.pone.0155863)
Supplement: S1 Table — The gender, age, ethnicity, occupation, location, diet style, BMI and blood oxygen level are listed for the 68 participants. (DOC) [file pone.0155863.s003.doc]

**S1 Table. Sampling Information. The gender, age, ethnicity, occupation, location, diet style, BMI and blood oxygen level are listed for the 68 participants.**

| **No.** | **Sex** | **Age (years)** | **Ethnicity** | **Occupation** | **Diet** | **Location** | **Altitude (m)** | **BMI**  **(kg/m^2^)** | **Blood oxygen saturation (%)** |
| --- | --- | --- | --- | --- | --- | --- | --- | --- | --- |
| 1 | M | 35 | Tibetan | Herder | Typical Tibetan pastoral | Tibet | 4500 | 23.5 | 85% |
| 2 | F | 39 | Tibetan | Herder | Typical Tibetan pastoral | Tibet | 4500 | 22.3 | 88% |
| 3 | M | 41 | Tibetan | Herder | Typical Tibetan pastoral | Tibet | 4500 | 23.9 | 84% |
| 4 | F | 50 | Tibetan | Herder | Typical Tibetan pastoral | Tibet | 4500 | 22.1 | 86% |
| 5 | F | 49 | Tibetan | Herder | Typical Tibetan pastoral | Tibet | 4500 | 19.2 | 85% |
| 6 | F | 50 | Tibetan | Herder | Typical Tibetan pastoral | Tibet | 4500 | 20.4 | 87% |
| 7 | M | 44 | Tibetan | Herder | Typical Tibetan pastoral | Tibet | 4500 | 23.2 | 84% |
| 8 | M | 43 | Tibetan | Herder | Typical Tibetan pastoral | Tibet | 4500 | 20.2 | 85% |
| 9 | M | 49 | Tibetan | Herder | Typical Tibetan pastoral | Tibet | 4500 | 21.5 | 84% |
| 10 | F | 40 | Tibetan | Herder | Typical Tibetan pastoral | Tibet | 4500 | 21.6 | 86% |
| 11 | M | 48 | Tibetan | Herder | Typical Tibetan pastoral | Tibet | 4500 | 23.4 | 85% |
| 12 | F | 37 | Tibetan | Herder | Typical Tibetan pastoral | Tibet | 4500 | 19.6 | 86% |
| 13 | M | 43 | Tibetan | Herder | Typical Tibetan pastoral | Tibet | 4500 | 23.8 | 83% |
| 14 | M | 50 | Tibetan | Farmer | Typical Tibetan | Tibet | 3600 | 22.7 | 92% |
| 15 | M | 38 | Tibetan | Farmer | Typical Tibetan | Tibet | 3600 | 23.1 | 91% |
| 16 | F | 42 | Tibetan | Farmer | Typical Tibetan | Tibet | 3600 | 22.2 | 91% |
| 17 | F | 48 | Tibetan | Farmer | Typical Tibetan | Tibet | 3600 | 23.8 | 90% |
| 18 | F | 49 | Tibetan | Farmer | Typical Tibetan | Tibet | 3600 | 20.3 | 91% |
| 19 | M | 47 | Tibetan | Farmer | Typical Tibetan | Tibet | 3600 | 21.5 | 92% |
| 20 | F | 38 | Tibetan | Farmer | Typical Tibetan | Tibet | 3600 | 23.4 | 90% |
| 21 | M | 50 | Tibetan | Farmer | Typical Tibetan | Tibet | 3600 | 20.5 | 91% |
| 22 | M | 43 | Tibetan | Farmer | Typical Tibetan | Tibet | 3600 | 20.3 | 89% |
| 23 | F | 36 | Tibetan | Farmer | Typical Tibetan | Tibet | 3600 | 21.4 | 90% |
| 24 | F | 49 | Tibetan | Farmer | Typical Tibetan | Tibet | 3600 | 23.6 | 91% |
| 25 | M | 42 | Tibetan | Farmer | Typical Tibetan | Tibet | 3600 | 20.8 | 92% |
| 26 | M | 47 | Tibetan | Farmer | Typical Tibetan | Tibet | 3600 | 23.5 | 91% |
| 27 | F | 36 | Han | Civil servant | Typical Han | Tibet | 3600 | 22.4 | 89% |
| 28 | M | 45 | Han | Civil servant | Typical Han | Tibet | 3600 | 23.2 | 88% |
| 29 | F | 50 | Han | Civil servant | Typical Han | Tibet | 3600 | 20.5 | 90% |
| 30 | M | 49 | Han | Civil servant | Typical Han | Tibet | 3600 | 19.4 | 89% |
| 31 | F | 48 | Han | Civil servant | Typical Han | Tibet | 3600 | 22.2 | 91% |
| 32 | F | 50 | Han | Civil servant | Typical Han | Tibet | 3600 | 23.2 | 89% |
| 33 | M | 43 | Han | Civil servant | Typical Han | Tibet | 3600 | 19.7 | 90% |
| 34 | M | 41 | Han | Civil servant | Typical Han | Tibet | 3600 | 21.6 | 90% |
| 35 | M | 40 | Han | Civil servant | Typical Han | Tibet | 3600 | 23.3 | 88% |
| 36 | F | 46 | Han | Civil servant | Typical Han | Tibet | 3600 | 23.0 | 90% |
| 37 | F | 39 | Han | Civil servant | Typical Han | Tibet | 3600 | 21.5 | 89% |
| 38 | M | 43 | Han | Civil servant | Typical Han | Tibet | 3600 | 20.1 | 90% |
| 39 | M | 39 | Han | Civil servant | Typical Han | Chengdu(Sichuan) | 500 | 20.5 | 97% |
| 40 | F | 42 | Han | Civil servant | Typical Han | Chengdu(Sichuan) | 500 | 22.3 | 98% |
| 41 | M | 50 | Han | Civil servant | Typical Han | Chengdu(Sichuan) | 500 | 21.6 | 98% |
| 42 | M | 36 | Han | Civil servant | Typical Han | Chengdu(Sichuan) | 500 | 19.9 | 98% |
| 43 | F | 37 | Han | Civil servant | Typical Han | Chengdu(Sichuan) | 500 | 21.0 | 99% |
| 44 | F | 47 | Han | Civil servant | Typical Han | Chengdu(Sichuan) | 500 | 20.8 | 98% |
| 45 | M | 45 | Han | Civil servant | Typical Han | Chengdu(Sichuan) | 500 | 23.5 | 97% |
| 46 | F | 46 | Han | Civil servant | Typical Han | Chengdu(Sichuan) | 500 | 22.4 | 99% |
| 47 | M | 42 | Han | Civil servant | Typical Han | Chengdu(Sichuan) | 500 | 20.6 | 99% |
| 48 | F | 39 | Han | Civil servant | Typical Han | Chengdu(Sichuan) | 500 | 23.4 | 98% |
| 49 | M | 50 | Han | Civil servant | Typical Han | Chengdu(Sichuan) | 500 | 19.6 | 97% |
| 50 | M | 49 | Han | Civil servant | Typical Han | Chengdu(Sichuan) | 500 | 21.9 | 98% |
| 51 | M | 44 | Han | Civil servant | Typical Han | Chengdu(Sichuan) | 500 | 19.6 | 99% |
| 52 | M | 38 | Han | Civil servant | Typical Han | Chengdu(Sichuan) | 500 | 23.3 | 100% |
| 53 | F | 46 | Han | Civil servant | Typical Han | Chengdu(Sichuan) | 500 | 21.8 | 97% |
| 54 | M | 44 | Han | Civil servant | Typical Han | Chengdu(Sichuan) | 500 | 22.4 | 98% |
| 55 | F | 36 | Han | Civil servant | Typical Han | Chengdu(Sichuan) | 500 | 20.8 | 98% |
| 56 | F | 49 | Han | Civil servant | Typical Han | Chengdu(Sichuan) | 500 | 22.1 | 96% |
| 57 | F | 36 | Han | Civil servant | Typical Han | Chengdu(Sichuan) | 500 | 19.4 | 98% |
| 58 | M | 45 | Han | Civil servant | Typical Han | Chengdu(Sichuan) | 500 | 22.5 | 99% |
| 59 | F | 44 | Han | Civil servant | Typical Han | Chengdu(Sichuan) | 500 | 21.4 | 98% |
| 60 | F | 47 | Han | Civil servant | Typical Han | Chengdu(Sichuan) | 500 | 22.0 | 99% |
| 61 | M | 37 | Han | Civil servant | Typical Han | Chengdu(Sichuan) | 500 | 21.3 | 98% |
| 62 | F | 43 | Han | Civil servant | Typical Han | Chengdu(Sichuan) | 500 | 19.7 | 97% |
| 63 | M | 45 | Han | Civil servant | Typical Han | Chengdu(Sichuan) | 500 | 23.5 | 98% |
| 64 | F | 50 | Han | Civil servant | Typical Han | Chengdu(Sichuan) | 500 | 20.4 | 97% |
| 65 | M | 48 | Han | Civil servant | Typical Han | Chengdu(Sichuan) | 500 | 21.6 | 99% |
| 66 | F | 38 | Han | Civil servant | Typical Han | Chengdu(Sichuan) | 500 | 22.1 | 96% |
| 67 | M | 44 | Han | Civil servant | Typical Han | Chengdu(Sichuan) | 500 | 23.4 | 98% |
| 68 | M | 36 | Han | Civil servant | Typical Han | Chengdu(Sichuan) | 500 | 22.8 | 99% |
